# Extracellular vesicles as a new hope for diagnosis and therapeutic intervention for hepatocellular carcinoma

**DOI:** 10.1002/cam4.4370

**Published:** 2021-10-28

**Authors:** Natthaphong Nimitrungtawee, Nakarin Inmutto, Siriporn C. Chattipakorn, Nipon Chattipakorn

**Affiliations:** ^1^ Diagnostic Radiology Unit Department of Radiology Faculty of Medicine Chiang Mai University Chiang Mai Thailand; ^2^ Cardiac Electrophysiology Research and Training Center Faculty of Medicine Chiang Mai University Chiang Mai Thailand; ^3^ Cardiac Electrophysiology Unit Department of Physiology Faculty of Medicine Chiang Mai University Chiang Mai Thailand; ^4^ Center of Excellence in Cardiac Electrophysiology Research Chiang Mai University Chiang Mai Thailand

**Keywords:** Biomarker, Diagnosis, Exosome, Extracellular vesicle, HCC, miRNA

## Abstract

Hepatocellular carcinoma (HCC) is the sixth most common cancer with a high mortality rate. Early diagnosis and treatment before tumor progression into an advanced stage is ideal. The current diagnosis of HCC is mainly based on imaging modalities such as ultrasound, computed tomography, and magnetic resonance imaging. These methods have some limitations including diagnosis in the case of very small tumors with atypical imaging patterns. Extracellular vesicles (EVs) are nanosized vesicles which have been shown to act as an important vector for cell‐to‐cell communication. In the past decade, EVs have been investigated with regard to their roles in HCC formation. Since these EVs contain biomolecular cargo such as nucleic acid, lipids, and proteins, it has been proposed that they could be a potential source of tumor biomarkers and a vector for therapeutic cargo. In this review, reports on the roles of HCC‐derived EVs in tumorigenesis, and clinical investigations using circulating EVs as a biomarker for HCC and their potential diagnostic roles have been comprehensively summarized and discussed. In addition, findings from in vitro and in vivo reports investigating the potential roles of EVs as therapeutic interventions are also presented. These findings regarding the potential benefits of EVs will encourage further investigations and may allow us to devise novel strategies using EVs in the early diagnosis as well as for treatment of HCC in the future.

## Graphical abstract

The roles of hepatocellular carcinoma (HCC)‐derived extracellular vesicles (EVs) in tumorigenesis, and clinical investigations using circulating EVs as a biomarker for HCC and their potential diagnostic roles have been comprehensively reviewed. The potential benefits of EVs from this review will encourage further investigations for the benefits of HCC patients.

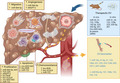

## 1 INTRODUCTION

Liver cancer is the sixth most common cancer found in cancer patients worldwide^1^ and 90% of liver cancer is hepatocellular carcinoma (HCC).^2^ Chronic liver injury from infection (hepatitis B or C viruses), chronic alcoholic intake, and fatty liver are well‐known risk factors for hepatocarcinogenesis.^3^ The treatments for HCC are dependent on tumor size, tumor location, and liver function. These interventions include tumor resection, local ablation, transarterial chemoembolization, and liver transplantation which are shown to be effective for HCC treatment.^4^, ^5^ However, some patients cannot be treated by these methods due to an advanced tumor stage and poor liver function.^6^ Currently, the overall 5‐year survival rate for HCC patients is still low despite the availability of multiple treatment modalities.^7^

Because most HCC patients are asymptomatic in the early stages, the early detection of small HCC before it progresses to the later stages is very important. Several pieces of evidence show that early detection of small HCC by screening with a liver ultrasound can significantly improve survival time.^8^ However, data from a meta‐analysis report showed that the sensitivity of ultrasound screening for detection of an early HCC is only 45%, and the sensitivity of combined ultrasound screening with serum alpha‐fetoprotein (AFP) for early HCC detection is only 63%.^9^ Currently, imaging such as CT and MRI are primary tools for the diagnosis of HCC in most guidelines.^10^, ^11^ With typical arterial enhancement and contrast washout in the delayed phase, HCC can be diagnosed without the need for tissue diagnosis.^12^ However, since approximately 40% of HCC patients showed atypical imaging features, this group of patients still need a liver biopsy for a definite diagnosis.^13^ All these findings indicate the need for improved tools to detect HCC earlier with increased sensitivity and specificity while being less invasive.

Liquid biopsy is one of the noninvasive methods for cancer diagnosis. Detection of circulating tumor DNA,^14^ circulating tumor cell,^15^ or extracellular vesicle (EV) in patients’ blood has been shown to be useful for cancer diagnosis. However, as the amount of circulating tumor cells and DNA are usually low in number with their short survival time in circulation, these factors, therefore, limit the use of these methods.^16^ The extracellular vesicle (EV) is a nano‐sized vesicle which can be produced and secreted by all cells.^17^ EVs can deliver molecules such as protein, mRNA, microRNA (miRNA), circularRNA (circRNA), long noncoding RNA (lncRNA), and DNA and play an important role in cell‐to‐cell communication.^17^ These vesicles can be found in almost all body fluid types such as blood, urine, ascites, effusion, and breast milk.^18^ There is increasing evidence to demonstrate that certain tumor cells can secrete EVs to modify the microenvironment of tumors and have a major role in tumor progression.^19^, ^20^

A previous study demonstrated that some liver‐derived transcripts can be found in the circulation of an HCC patient.^21^ The lipid raft domain structure of EVs can protect these transcripts against degradation by circulating RNase, thus allowing the EV‐RNA to be stable in the blood.^22^ As a result, these EVs have been proposed as a potential diagnostic biomarker.^22^, ^23^, ^24^ Furthermore, it has been shown that EVs could be used to stimulate an immune response as well as to carry the tumor suppressor gene, all of which could have therapeutic potential in cancer treatment.^25^, ^26^ In this review, the biologic roles of HCC‐derived EVs obtained from in vitro and in vivo reports are comprehensively summarized. Reports on their potential roles as diagnostic biomarkers and possible therapeutic interventions are also presented and discussed.

Literature was searched on the PubMed database from its inception until August 30, 2021 using the following search terms: hepatocellular carcinoma, extracellular vesicle, and exosome. In vitro, in vivo, and clinical studies were included in this review.

## 2 BIOLOGIC FUNCTION OF HCC CELL‐DERIVED EVS: REPORTS FROM IN VITRO STUDIES

Tumor microenvironments play an essential role in HCC development and progression.^27^, ^28^ Recent evidence showed the dysregulation of multiple signaling pathways and cell‐to‐cell communication in HCC.^29^ HCC‐derived EVs are one of the major mediators of cell‐to‐cell communication.^30^ They were found to contain multiple miRNAs, lncRNA, and circRNA which regulate tumor cell proliferation,^31^, ^32^, ^33^, ^34^, ^35^, ^36^, ^37^ migration,^33^ and decrease tumor cell apoptosis and chemoresistance.^32^, ^36^, ^37^ Several RNAs can regulate the tumor microenvironment by increasing angiogenesis and decreasing cell adhesion.^31^, ^33^, ^34^ In addition, serum‐free miRNA in cancer patients has been shown to correlate with tumor cell proliferation, metabolism, invasion, and metastasis.^38^ Recent evidence showed that other noncoding RNA (lncRNA and circRNA) are also related to tumorigenesis. CircRNA has been demonstrated to act as an miRNA sponge,^39^ whereas the dysregulation of lncRNA was associated with cancer.^40^ High metastatic HCC cell (LM3) had increased expression of EV‐circ‐PTGR1 which has been shown to increase cell migration and apoptosis.^41^ HepG2 and HuH7 cell lines also had increased EV‐lnc 544, 239, 959, 171, and 85 and were shown to increase cell proliferation.^42^ As these molecules in HCC‐derived EVs promoted the survival of the tumor cells, they could be a target for HCC treatment. A summary of these reports from in vitro studies is shown in Table 1.

**TABLE 1:** Biologic functions of HCC cell‐derived EVs: Reports from in vitro studies

HCC cell line	EV extraction method	EV molecule expression	Major findings (tumor cells)					Interpretation	References
			Proliferation	Apoptosis	Migration	Chemoresistance	Microenvironment		
Hep3B	DC, UF	↑ miR−584‐5p	↑		↑		↑ Angiogenesis	miR−584‐5p increased HCC cell proliferation, migration, and angiogenesis	^33^
Hep3B, HuH7	DC, Exoquick	↓ circ−0051443	↑	↓ Bak1				HCC cell showed decreased circ−0051443 which acts as a tumor suppressor gene	^32^
Hep3B, HepG2, PLC/PRF/5	DC, DGC	↑ TUC−339	↑				↓ HCC cell adhesion	TUC−339 increased HCC cell proliferation and might increase invasion/metastasis	^34^
Hep3B, SNU18, SNU38, Li7, and MHCC97H	DC	↑ ANGPT−2	↑				↑ Angiogenesis	ANGPT−2 increased HCC cell proliferation and angiogenesis	^31^
MHCC−97L, MHCC−97H	DC, Exoquick	↑ miR−665	↑					miR−665 increased HCC cell proliferation	^35^
HUH7	DC, UF	↑ miR−122	↓	↑ cleaved PARP, Caspase 9		↓ (Doxorubicin)		HUH7 cell (less‐aggressive cell line) had high miR−122 which decreased HCC cell proliferation, increased apoptosis, and decreased chemoresistance of tumor cell	^88^
HepG2	UC	↑ linc‐VLDLR	↑			↑ (Sorafenib, Doxorubicin)		linc‐VLDLR increased HCC cell proliferation and chemoresistance	^36^
HepG2, PLC‐PRF5	DC	↑ linc‐ROR	↑	↓ (Caspase 3/7)		↑ (Sorafenib, Doxorubicin, Camptothecin)		linc‐ROR increased HCC cell proliferation, chemoresistance and decreased HCC cell apoptosis	^37^
HepG2, HuH7	Ribo exosome isolation reagent	↑lnc 544	↑	↓	↔			Lnc−85, −171, −959, −239, −554 increased HCC cell proliferation Lnc−85, −171, −544 decreased HCC cell apoptosis Lnc−85, −959, −239 increased HCC cell migration	^42^
		↑lnc 239	↑	↔	↑				
		↑lnc 959	↑	↔	↑				
		↑lnc 171	↑	↓↓	↔				
		↑lnc 85	↑↑	↓↓	↑				
97hm, Huhm	UC	↑ miR−92a−3p			↑		↑ Metastasis	miR−92a−3p increased HCC cell migration and metastasis	^89^
LM3	Exoquick	↑ circ‐PTGR1		↓	↑			High metastatic HCC cell (LM3) had increased EV‐circ‐PTGR1 and was associated with increased cell migration and decreased apoptosis	^41^

Abbreviations: ABC, ATP‐binding cassette; ANGPT, angiopoietin; circ‐RNA, circular RNA; DC, differential centrifugation; DGC, density‐gradient separation; IGF, insulin growth factor; linc‐ROR, long intergenic noncoding RNA; lnc‐RNA, long noncoding RNA; omiR, oncogenic microRNA; tsmiR, tumor suppressor miRNATUC siRNA, ultraconserved long noncoding siRNA; TUC, tumor ultraconserved RNA; UF, ultrafiltration.

## 3 CIRCULATING EV MIRNA IN HCC PATIENTS: REPORTS FROM CLINICAL STUDIES

In the last decades, multiple studies have reported an association between serum circulating miRNA and HCC including miR‐15b, miR‐16, miR‐19a, miR‐21, miR‐27b, miR‐92a, miR‐107p, miR‐122, miR‐130b, miR‐183, miR‐192, miR‐195, miR‐221, miR‐222, miR‐223, miR‐224, and ETC.^43^, ^44^, ^45^, ^46^, ^47^, ^48^, ^49^, ^50^, ^51^, ^52^, ^53^, ^54^ Recent evidence has also shown the superiority of EV‐miRNA over serum biomarkers in accurate diagnosis.^55^, ^56^, ^57^ Since EVs selectively pack and carry specific cargo (i.e., protein, DNA, RNA, and tumor‐specific transcripts) from their cell of origin, it is proposed that the cargos in EVs are potential diagnostic biomarkers.^58^, ^59^, ^60^

EVs can be secreted by all cell types including HCC cells^61^ and can be detected in bodily fluids, especially in the blood.^18^ Unlike RNA in the serum, RNA inside the EV can be protected from RNase and other adverse conditions in circulation making EV‐RNA more stable in the blood. EV‐RNA can also be used as a diagnostic biomarker for tumors.^23^ Moreover, EV‐RNA can also be quantitated by qRT‐PCR, thus making their analysis comparable with conventional proteomic methods.^62^ Currently, there are multiple methods to extract EVs from plasma by using their physical properties including ultracentrifugation, filtration, size exclusion chromatography, and precipitation. Ultracentrifugation (UC) is the commonly used method; however, it is burdensome and is unlikely to be used in clinical practice.^63^ The filtration method uses less time compared with the UC, however, some EVs may be lost due to the jamming of EVs on the filter.^64^ Size exclusion chromatography has been shown to effectively isolate protein contaminants from EVs; however, it has relatively low throughput.^65^ Precipitation with UC is a time‐saver and can be used to test multiple concurrent samples simultaneously.^66^ However, some non‐EV contaminants will also be obtained. Recently, Sun et al. had developed the novel EV purification system, EV Click chips.^67^ This method combined covalent chemistry‐mediated EV capture/release, multimarker antibody cocktails, nanostructured substrates, and microfluidic chaotic mixers to selectively extract tumor‐derived EV from total circulating EV. This method has been shown to overcome the limitation of prior methods for EV extraction and was proposed to increase the diagnostic power of circulating EV.^67^

In the majority of reports included in this review, a combination of a UC method and other methods were frequently used. However, despite almost all studies used the qRT‐PCR technique to measure the EV content (Table 2), one used the TLN biochip combined with TIRF microscopy to detect small fragments of mRNA.^55^ This method might be more effective than qRT‐PCR which can detect only intact large fragments of mRNA.

**TABLE 2:** Circulating EV miRNA in HCC patients

Subjects (N)	EV source	EV extraction method	Molecule detection method	EV biomarker expression	Clinical relevance	Interpretation	Reference
HCC (48) LC (38) Healthy controls (20)	Plasma (5 ml)	TEIp kit (DC, PEG)	qRT‐PCR	HCC ↑ miR−21‐5p ↓ miR−92a−3p		HCC patients had increased exosomal miR−21‐5p and decreased miR−92a−3p in comparison with the cirrhotic patient.	^56^
				LC ↔ miR−21‐5p ↔ miR−92a−3p			
HCC (50) CHB (40) LC (40) Healthy controls (64)	Serum	DC, PEG	miRNeasy mini kit +qRT‐PCR	HCC, CHB ↑ miR−122, −148a, −1246		HCC and CHB patients had increased exosomal miR−122, 148a, 1246 None of these biomarkers could be used to differentiate HCC from CH	^57^
				LC ↔ miR−122, −148a, −1246			
HCC (86) CCA (38) LC (54) Healthy controls (202)	Serum (7.5 ml)	DC	FACS	HCC & CCA ↑↑ AnnexinV^+^ EpCAM^+^ MV ↑↑ AnnexinV^+^ EpCAM^+^ ASGPR1^+^ MV	AnnexinV^+^ EpCAM^+^ MV correlates with tumor size	Circulating AnnexinV^+^ EpCAM^+^ and AnnexinV^+^ EpCAM^+^ ASGPR1^+^ MV increased in HCC patients	^74^
				LC ↑ AnnexinV^+^ EpCAM^+^ MV ↔ AnnexinV^+^ EpCAM^+^ ASGPR1^+^MV			
HCC (71) Healthy controls (32)	Plasma	N/A	qRT‐PCR	↑ TST1	↓ TST1 after curative surgery	Presence of TST1 correlated with HCC	^60^
HCC (40) Healthy controls (38)	Plasma (6 ml)	DC, Exoquick	qRT‐PCR vs TLN biochip +TIRF microscopy	↑ AFP mRNA (AFP−174, MB−1096, MB−1171) ↑ GPC3 mRNA (GPC3 MB)		Increased exosomal AFP mRNA and GPC3 mRNA in HCC patients	^55^
HCC (30) Healthy controls (10)	Serum	DC, Exoquick	qRT‐PCR	HCC ↑ miR−665	↑ miR−665 (>5 fold) correlate with higher stage, larger tumor size (>5 cm), and metastasis	HCC patient had significantly increased serum exosomal miR−665 and it can be a prognostic marker	^35^
HCC (30) CHB (30) Healthy controls (30)	Serum exosome	DC, Isolation agent (Invitrogen)	qRT‐PCR	HCC (serum exosome) ↑↑↑ miR−21 HCC (whole serum) ↑↑ miR−21	↑ miR−21 in HCC patient correlate with tumor stage	Exosomes increased sensitivity of miRNA detection in serum Exosomal miR−21 is increased in HCC and CHB patients	^68^
				CHB (serum exosome) ↑↑ miR−21 CHB (serum exosome) ↑ miR−21			
HCC (59)	Serum	DC, UF	qRT‐PCR	↑ miR−1246 ↓ miR−718 (tumor >3 cm)	↓ miR−718 correlate with tumor size (>3 cm) and number of tumor	In HCC patients, exosomal miR−1246 was increased and miR−718 was decreased	^62^
HCC (20) LC (20) CHB control (20)	Serum exosome (0.5 ml)	Exoquick	qRT‐PCR	HCC (serum exosome) ↑ miR−18a, −221, −222, −224 ↔ miR−21, −93 ↓ miR−101, −106b, −122, −195 HCC (whole serum) ↔ miR−21, −101, −195, −221, −222, −224		Serum exosomal microRNAs was more effective in distinguishing HCC from CHB and LC compared with whole serum circulating microRNAs	^69^
				LC (serum exosome) ↔ miR−18a, −21, −93, −101, −106b, −122, −195, −221, −222, −224			
HCC (90) CHB (28) LC (35) Normal (29)	Serum (5 ml)	Exoquick	qRT‐PCR	HCC ↑ lncRNAs (LINC00853, SFTA1P, HOTTIP, HAGLROS, LINC01419, HAGLR, CRNDE)	LINC00853 elevated in AFP‐negative HCC Patients with high EV‐LINC00853 had a lower survival rate	HCC patients had increased EV‐lncRNAs. LINC00853 might be used for HCC diagnosis, especially in AFP‐negative HCC. It was also correlated with patients’ prognosis	^70^
				LC, CHB, Normal ↔ lncRNAs (LINC00853, SFTA1P, HOTTIP, HAGLROS, LINC01419, HAGLR, CRNDE)			
HCC (46) CHB (25) LC (26) Normal (23) Liver metastasis (12) Other primary cancer (26)	Plasma	EV click chips	RT‐ddPCR	HCC ↑ GPC3, AFP, AHSG, TF ↑↑ ALB, APOH, FABP1, FGB, FGG, RBP4	FGG, FGB, and RBP4 showed increased expression in HCC‐BCLC stage B‐C compared with stage 0‐A	EV click chips could selectively purify EV from HCC cells. These 10 EV‐mRNA were increased in HCC patients, compared with CHB, LC, liver metastasis, other primary cancer, and normal control	^67^
				CHB ↔ GPC3, AFP, FABP1 ↑ AHSG, APOH, FGB, FGG, RBP4 ↑↑ ALB			
				LC, Normal, Liver metastasis, Other cancer ↔ GPC3. AFP, AHSG, APOH, FABP1, FGB, FGG, RBP4, TF ↑ ALB			
HCC (38) Chronic hepatitis (35) Liver cirrhosis (25) Normal (11)	Plasma	ExoEnrich	qRT‐PCR	HCC ↑↑ miR21‐5p ↑ miR10b−5p, miR221‐3p, mir223‐3p		miR 21‐5p showed significantly increased expression in HCC compared with chronic hepatitis and LC patients	^71^
				Chronic hepatitis and LC ↑ miR10b−5p, miR221‐3p, mir223‐3p, mir21‐5p			
HCC (71) Normal (40)	Plasma	UC	qRT‐PCR	HCC ↑ hsa‐circ−0004001, 0004123, 0075792	Hsa‐circ−0004001 and 0075792 were significantly associated with TNM tumor staging while hsa‐circ−0004001 and 0004123 were associated with tumor size.	EV‐hsa‐circ−0004001, 0004123, 0075792 were increased in HCC patients	^73^
Early stage HCC (50) Normal (100)	Plasma	ExoRNeasy	qRT‐PCR	HCC ↑ LDHC‐mRNA	LDHC‐mRNA was associated with survival outcome	EV‐LDHC‐mRNA expression was increased in early stage HCC and was associated with survival outcome	^72^

Abbreviations: AUC, area under the curve; BCLC, Barcelona clinic liver cancer; CCA, cholangiocarcinoma; CHB, chronic hepatitis B; circ, circular RNA; DC, differential centrifugation; FACS, fluorescence‐activated cell scanning; HCC, hepatocellular carcinoma; LC, liver cirrhosis; lnc‐RNA, long noncoding RNA; MV, microvesicle; PEG, polyethylene glycol precipitation; TEIp, total exosome isolation; TIRF, total internal reflective fluorescence; TLN, tethered lipoplex nanoparticles; TST, tumor‐specific transcript; UF, ultrafiltration.

Many clinical studies have shown alterations in circulating EV‐RNA in HCC patients. Wang et al. and Sohn et al. investigated both EV‐miRNA and serum‐free miRNA. They proved that miRNA detection in EVs can result in increased sensitivity compared with serum‐free miRNA.^68^, ^69^ Seven reports found a significant difference in circulating EV‐RNA in HCC patients compared with LC or CHB patients which represent real‐life situations.^56^, ^67^, ^68^, ^69^, ^70^, ^71^, ^72^ Moreover, multiple EV‐mRNA, miRNA, lncRNA, and circRNA have been shown to correlate with tumor burden.^35^, ^62^, ^67^, ^68^, ^72^, ^73^ In addition, EV surface antigens (Annexin V, EpCAM, and ASGPR1) were also detected in the serum of HCC and CCA patients.^74^ Reports on these findings are summarized in Table 2.

## 4 EV AS A DIAGNOSTIC TEST: EVIDENCE FROM CLINICAL REPORTS

In the past decade, there are several studies which used a combination of multiple circulating EV‐RNA or EV‐surface antigens to develop diagnostic tests for HCC. There are five reports of high diagnostic performance model scores for diagnosing HCC in liver cirrhosis patients.^42^, ^56^, ^57^, ^67^, ^70^ Combined *Z*‐score of 10 EV‐mRNA (ALB, GPC3, AFP, AHSG, APOH, FABP1, FGB, FGG, RBP4, and TF) showed high diagnostic performance for HCC diagnosis from at‐risk patients (sensitivity 93.8%, specificity 74.5%, AUC 0.87) and other primary cancer (sensitivity 95.7%, specificity 89.5%, AUC 0.95).^67^ High ability for early HCC detection from liver cirrhosis patients was also demonstrated (sensitivity 94.4%, specificity 88.5%, AUC 0.93).^67^ One report used a combination of serum EV‐miR‐21‐5p, EV‐miR‐92a‐3p, and AFP which showed a sensitivity of 95% with a specificity of 50%, and an AUC of 0.85.^56^, ^57^ The other report used a combination of serum EV‐miR‐122, EV‐miR‐148a, and AFP which resulted in a sensitivity of 86%, specificity of 87.5%, and AUC of 0.93.^56^, ^57^ A better diagnostic performance using a combination of model scores was demonstrated compared with conventional biomarkers (AFP and GPC3) in HCC patients.^55^, ^56^

Recently, two reports using the EV‐lncRNA method have demonstrated that it could provide higher diagnostic performance than serum AFP.^42^, ^70^ EV‐LINC00853 demonstrated the sensitivity of 93.75% with a specificity of 89.77% and AUC of 0.97 for the diagnosis of early HCC in patients at‐risk group (liver cirrhosis and hepatitis B).^70^ In addition, EV‐lnc85 showed a high diagnostic performance for differentiating both AFP‐positive (AUC 0.90) and AFP‐negative HCC (AUC 0.88) from liver cirrhosis patients.^42^

In cirrhosis patients, in addition to serum EV‐RNA, the EV surface antigen has been investigated for its diagnostic potential of HCC and CCA.^74^ Although an increase in serum EV surface antigens was found in HCC and CCA patients,^74^ the results indicated that EV surface antigens could not be used to differentiate HCC from CCA patients.^74^ In HCC patients, another report using tumor‐specific transcript‐1 (TST‐1) demonstrated its very high specificity (100%) for HCC detection but low sensitivity (28%).^60^ These reports are summarized in Table 3.

**TABLE 3:** EV as a potential diagnostic marker: Reports from clinical studies

Subjects (N)	EV source	EV extraction method	Molecule Detection method	Index test	Aim of test	Diagnostic indices			Interpretation	References
						Sensitivity (%)	Specificity (%)	AUC		
HCC (48) LC (38) Control (20)	Plasma (5 ml)	TEIp kit (DC, PEG)	qRT‐PCR	Model score of miR−21‐5p + miR−92a−3p + AFP	Dx HCC from cirrhosis	95	50	0.85	Model score of miR−21‐5p + miR−92a−3p + AFP had high accuracy for diagnosis of HCC in cirrhosis	^56^
HCC (50) CHB (40) LC (40) Control (64)	Serum	DC, PEG	miRNeasy mini kit +qRT‐PCR	Model score of miR−122 + 148a + AFP	Dx HCC from cirrhosis	86	87.5	0.93	This test could be used in LC patients without hepatitis B infection	^57^
HCC (86) CCA (38) LC (54) Control (202)	Serum (7.5 ml)	DC	FACS	AnnexinV^+^ EpCAM^+^ ASGPR1^+^ taMP (4.2 fold rising)	Dx liver tumor from cirrhosis	75	47	0.7	Index test could not distinguish between HCC and CCA	^74^
HCC (71) Control (32)	Plasma	N/A	qRT‐PCR	TST1	Dx HCC vs. healthy controls	28	100	–	Presence of TST1 had very high specificity for HCC diagnosis	^60^
HCC (40) Control (38)	Plasma (6 ml)	DC, Exoquick	qRT‐PCR vs. TLN biochip +TIRF microscopy	Combined AFP mRNA +GPC3 mRNA	Dx HCC vs. healthy controls	95	100	0.99	Combined AFP mRNA +GPC3 mRNA had high accuracy for diagnosis of HCC	^55^
HCC (90) CHB (28) LC (35) Normal (29)	Serum (5 ml)	Exoquick	qRT‐PCR	EV‐LINC00853	Dx early HCC from cirrhosis and chronic hepatitis B	93.75	89.77	0.97	Ev‐LINC00853 level had high accuracy for HCC diagnosis in cirrhosis	^70^
HCC (112) LC (43) Normal (52)	Plasma	Ribo exosome isolation reagent	qRT‐PCR	EV‐lnc85	Dx HCC from cirrhosis and normal	80.0	74.5	0.87	EV‐lnc85 level had high accuracy for HCC diagnosis in both AFP‐negative and AFP‐positive HCC	^42^
					Dx HCC from cirrhosis	80.0	74.4	0.89		
					Dx AFP‐positive HCC from cirrhosis	80.5	76.7	0.90		
					Dx AFP‐negative HCC from cirrhosis	80.0	76.7	0.88		
HCC (46) CHB (25) LC (26) Normal (23) Liver metastasis (12) Other primary cancer (26)	Plasma	EV‐click chips	RT‐ddPCR	Combined Z‐score of 10 mRNA (ALB,GPC3. AFP, AHSG, APOH, FABP1, FGB, FGG, RBP4, TF)	Dx HCC from noncancer (CHB, LC)	93.8	74.5	0.87	Combined *Z*‐score of mRNA had high accuracy for differentiating HCC from noncancer, other primary cancer and liver cirrhosis patients	^67^
					Dx HCC from other primary cancer	95.7	89.5	0.95		
					Dx early HCC from liver cirrhosis	94.4	88.5	0.93		
HCC (38) CHB or CHC (35) Liver cirrhosis (25) Normal (11)	Plasma	ExoEnrich	qRT‐PCR	Model score of 4 miRNA (miR10b−5p, miR221‐3p, mir223‐3p, mir21‐5p)	Dx HCC from LC and hepatitis	58.0	95.0	0.80	Model score of four miRNA showed high specificity for HCC diagnosis, but its sensitivity was limited	^71^
HCC (71) Normal (40)	Plasma	UC	qRT‐PCR	Model score of 3 circRNAs (hsa_circ_0004001, 0004123, 0075792)	Dx HCC from normal control	90.5	78.1	0.89	Model score of three circRNAs had high diagnostic accuracy for HCC diagnosis from normal control	^73^
Early stage HCC (50) Normal (100)	Plasma	ExoRNeasy	qRT‐PCR	EV‐LDHC‐mRNA	Dx early stage HCC from normal control	88.2	93.3	0.95	EV‐LDHC‐mRNA had high diagnostic accuracy for early HCC diagnosis from normal control	^72^

Abbreviations: AUC, area under the curve; CCA, cholangiocarcinoma; CHB, chronic hepatitis B; CHC, chronic hepatitis C; DC, differential centrifugation; HCC, hepatocellular carcinoma; LC, liver cirrhosis; MV, Microvesicle; PEG, polyethylene glycol precipitation; TEIp, total exosome isolationTIRF, total internal reflective fluorescence; TLN, tethered lipoplex nanoparticles; TST, tumor‐specific transcript.

## 5 EV AS A THERAPEUTIC INTERVENTION FOR HCC: EVIDENCE FROM IN VITRO REPORTS

EVs can be loaded with therapeutic cargo such as miRNA enabling transference into the target tumor cell. EVs may therefore be used as a personalized cancer treatment.^75^ There are several in vitro studies using EVs for HCC cell treatment. The sources of EVs vary, examples being hepatic stellate cells, stem cells, HCC cells, hepatocytes, and bovine fat‐free milk. The majority of studies used an endogenous loading method to load miRNA or other therapeutic molecules into EVs. The endogenous loading method (preloading) is to modify the target donor cells before EV shedding.^76^, ^77^ After loading therapeutic molecules to the donor cells, the donor and recipient HCC cells are cocultured enabling EV transference into the recipient HCC cell. All studies have shown that their therapeutic cargo can be transferred to target tumor cells and increase cell apoptosis,^78^ decrease chemoresistance,^79^ reduce cell proliferation,^78^, ^80^, ^81^, ^82^ and reduce cell migration.^80^

In another report, sodium iodide symporter (NIS) genes were transfected to donor HCC cells and cocultured with recipient HCC cells.^83^ The NIS protein increased the I‐131 toxicity in recipient HCC cells, thus allowing the HCC cells to be susceptible to I‐131 ablation.^83^ Most studies use EVs as a therapeutic cargo.^78^, ^79^, ^80^, ^81^ In one study, EVs from tumor cells were used to activate bone marrow stem cells.^82^ Then, these activated bone marrow stem cells were cocultured with tumor cells. The results showed that tumor proliferation was significantly decreased. This method is known as the “Exosome‐based vaccine”.^82^ All these in vitro reports are summarized in Table 4.

**TABLE 4:** EV as a potential therapeutic intervention for HCC: Evidence from in vitro reports

Molecule	Donor cell	Recipient cell	EV extraction method	TEV	TEV using method	Major Findings (Tumor cell)				Interpretation	References
						Proliferation	Apoptosis	Migration	Chemoresistance		
miR335‐5p	HSC cell (LX2)	HCC cell (MHCC97H, MHCC97L, HepG2, Huh7)	DC	HSC‐EV‐miR335‐5p (EL)	Coculture	↓		↓		HSC‐EV‐ miR335‐5p (EL) decreased tumor cell proliferation and migration	^80^
miR125b	ASC cell	HCC cell (HuH7, HepG2)	Exoquick	ASC‐EV‐miR125b (EL)	Coculture	↓				Human adipose cell might be useful as a source of therapeutic EVs	^81^
miR451, 223, 24, 125b, and 31	HLSC	HCC cell (HepG2)	DC	HLSC‐MV (contained miR451, 223, 24, 125b, and 31)	Coculture	↓	↑ (TUNEL assay)			HLSC‐MV decreased tumor cell proliferation and increased apoptosis	^78^
BMSC^TEX,IFN− γ^ (exosome based tumor vaccine)	Mouse HCC cell (H22)	Murine BMSC	DC, UF	TEX from H22 cell	Coculture HCC cell with TEX or unactivated BMSC or IFN‐ γ	↔				Exosome‐based vaccine was a potential way to treat HCC	^82^
					Use TEX +IFN‐γ to activate BMSC then coculture it with H22 cell	↓					
miR−122	AMSC	HCC cell (HepG2, HuH7)	Exoquick	AMSC‐EV‐miR−122 (EL)	Coculture	↔	↔ (Annexin V, PI)			AMSC‐EV‐miR−122 (EL) treatment alone had no effect on HCC cell but increased the effect of chemotherapy	^79^
					Give sorafenib or 5‐FU	↓	↑ (Annexin V, PI)				
					Coculture and give sorafenib or 5‐FU	↓↓	↑↑ (Annexin V, PI)		↓		
NIS protein	HCC cell (HuH7)	HCC cell (HuH7)	DC	HCC‐EV ‐NIS gene (EL)	Coculture and give I−131					NIS protein transferred by EV increased cytotoxicity and DNA damage of I−131 to HCC cell	^83^
miR150‐3p	Normal fibroblast (NF)	HCC cell (HuH7, Hep3B)	Total exosome isolation reagent	NF‐EV‐miR150‐3p (EL)	Coculture			↓		NF‐EV‐ miR150‐3p (EL) decreased tumor cell migration	^90^

**Abbreviations**: ANGPT, angiopoietin; AMSC, adipose mesenchymal stem cell; ASC, human adipose stem cell; BMSC, bone marrow stem cell; CAF, cancer‐associated fibroblast; DC, differential centrifugation; EL, endogenous loaded; HCC, hepatocellular carcinoma; HLSC, human liver stem cell; HSC, hepatocyte stellate cell; HUVEC, human umbilical vein endothelial cell; I‐131, iodine isotope 131; IFN, interferon; Linc, long intergenic noncoding RNA; NIS, sodium iodide symporter; TDEV, tumor‐derived EV; TEV, therapeutic EV; TEX, tumor‐derived exosome; UF, ultrafiltration.

## 6 EV AS A THERAPEUTIC INTERVENTION FOR HCC: EVIDENCE FROM IN VIVO REPORTS

In this context, most in vivo studies were done in an immunocompromised mouse model such as NOD SCID mice or nude mice in which an HCC xenograft tumor was subcutaneously implanted to these mice (Table 5). Therapeutic molecules which had antitumoral effects from in vitro studies were endogenously loaded into EVs. The route for EVs treatment introduction was mainly either intratumoral injections or intravenous injections via tail veins. The therapeutic EVs resulted in decreased tumor sizes,^32^, ^78^, ^79^, ^80^, ^84^, ^85^, ^86^, ^87^ increased tumor cell apoptosis,^32^, ^78^, ^79^, ^80^ and increased chemoresistance.^79^, ^84^

**TABLE 5:** EV as a therapeutic intervention for HCC: Evidence from In Vivo Reports

Model (N)	Xenograft cell line	Donor cell	Molecule	TEV	Route/Dose/Duration	Major findings			Interpretation	References
						Tumor volume	Apoptosis	Chemoresistance		
Female NOD SCID gamma mice	HCC cell (MHCC97H)	HSC cell (LX2)	miR335‐5p	LX2‐EV‐miR−335	IT/50 μg exosome two times/week/4 weeks	↓	↑ (Caspase 3)		IT LX2‐EV‐miR−335 decreased tumor volume and increased apoptosis of xenograft HCC	^80^
Male nude mice	HCC cell (HuH7)	Hepatocyte (HL7702)	Circ−0051443	HL7702‐EV‐circ−0051443	IT/10 μg exosome OD/15 days	↓	↑ (BAK1 expression)		IT HL7702‐EV‐circ−0051443 decreased tumor volume and increased apoptosis of xenograft HCC	^32^
Friend virus B mice (21)	Induce HCC by coactivation of cMET and β‐catenin mutation	Bovine fat‐free milk	β‐catenin siRNA	MNV‐loaded siRNA β‐catenin	IV/TEV 2 x 10^12^ particles/body every 3 days/5 doses	↓			Fat‐free milk can be used as a source of EV MNV loaded siRNA β‐catenin decreased tumor size and chemoresistance (anti‐PD−1)	^84^
					IP/250 μg anti‐PD−1 three times/week/2 weeks	↓↓				
					Combined IV TEV and IP anti‐PD−1/2 weeks	↓↓↓		↓		
Athymic nude mice	HCC cell (LCSC, Hep3B)	Bovine fat‐free milk	β‐catenin siRNA	ET‐tMNV‐loaded siRNA β‐catenin	IV/tMNV 5 × 10^10^ particles/body every 2 days/5 doses	↓			ET‐tMNV targeted EpCAM‐expression cells and decreased xenograft HCC growth	^85^
Male C57L/J mice (40)	Mouse HCC cell (Hepa 1–6)	Mouse macrophage cell line (raw 264.7)	miR−142‐3p	TAM‐EV‐miR−142‐3p (stimulated by propofol)	IP/propofol 20 or 50 mg/kg every day/3 weeks	↓			Propofol decreased HCC growth by activating TAM to produce EV‐miR−142‐3p	^86^
C57BL/6 wild‐type mice (10), BALB/C nude mice (20)	Mouse HCC cell (Hepa 1–6)	Mouse HCC cell (Hepa 1–6)	DCC^TEX^ (exosome‐based tumor vaccine)	Use TEX to activated DCC (DCC^TEX^)	IV/DCC^TEX^ 2 × 10^6^ one dose every 2 weeks/three doses	↓			Tumor exosome activated DCC. The activated DCC^TEX^ decreased tumor volume	^87^
Nude mice (10)	HCC cell (HepG2)	AMSC	miR−122	AMSC‐EV‐miR−122	Single‐dose IT/AMSC‐EV‐miR−122 50 μg with IP sorafenib (5 mg/kg) five doses/week/5 weeks	↓	↑ (Caspase 3, Bax)	↓	AMSC‐EV‐miR−122 alone had no effect on xenograft HCC, but it decreased chemoresistance	^79^
					Single‐dose IT/AMSC‐EV‐miR−122 50 μg	↔	↔			
Male SCID mice	HCC cell (HepG2)	HLSC	miR451, 223, 24, 125b, and 31	HLSC‐MV	IT/HLSC‐MV 100 μg/20 μl weekly/3 weeks	↓	↑ (TUNEL assay)		HLSC‐MV decreased HCC growth and increased apoptosis	^78^

**Abbreviations**: AMSC, adipose mesenchymal stem cell; CAF, cancer‐associated fibroblast; circ, circular RNA; cMET, c‐tyrosine‐protein kinase MET; DC, differential centrifugation; DCC, dendritic cell; EpCAM, epithelium cell adhesion molecule; ET, EpCAM targeted; HCC, hepatocellular carcinoma; HLSC, human liver stem cell; HSC, hepatocyte stellate cell; IP, intraperitoneal injection; IT, intratumoral injection; IV, intravenous injection; LCSC, liver cancer stem cell; MNV, milk‐derived nanovesicle; SC, subcutaneous injection; TAM, tumor‐associated macrophage; TDEV, tumor‐derived EV; TEV, therapeutic EV; TEX, tumor‐derived exosome; tMNV, therapeutic milk‐derived nanovesicle; UC, ultra centrifugation.

In addition to using EVs as a therapeutic cargo, indirect use regarding the efficacy of EVs for HCC treatment has been reported. Intraperitoneal injection of propofol was shown to stimulate tumor‐associated macrophages to produce miR‐142‐3p EVs, which was associated with decreased tumor growth.^86^ In one study, an exosome‐based tumor vaccine (dendritic cells stimulated by tumor EVs) was used to treat xenograft HCC in mice.^87^ The result showed that intravenous injection of exosome‐based tumor vaccine effectively decreased xenograft tumor volume.^87^ All these in vivo studies demonstrated the benefit of EVs as a potential tool for HCC treatment including the use of EVs as a therapeutic cargo or a tumor vaccine since EV could transfer therapeutic molecules into the xenograft HCC, leading to decreased tumor proliferation. These reports are summarized in Table 5.

## 7 CONCLUSION AND FUTURE PROSPECTIVE

**FIGURE 1:**
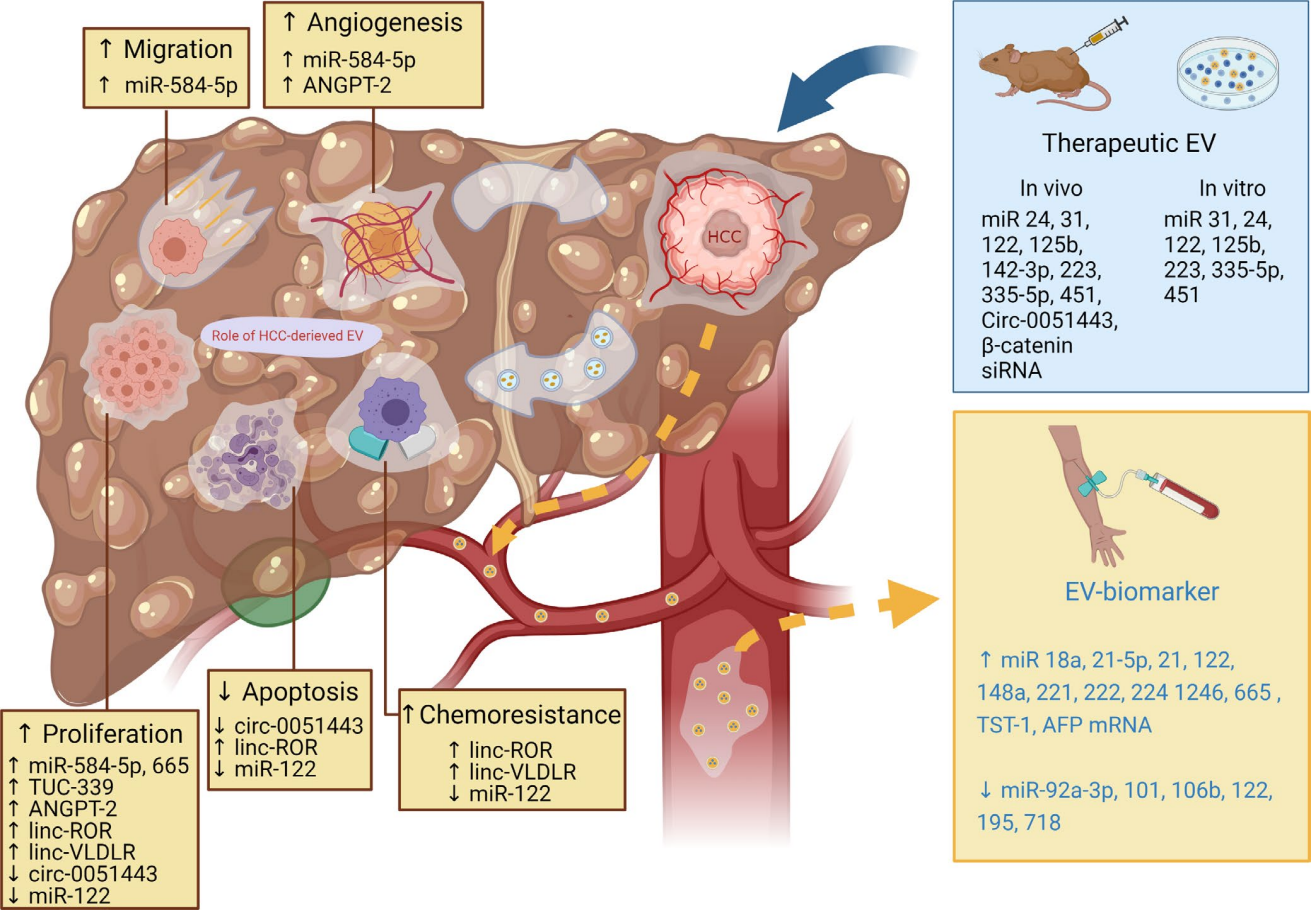
The roles of HCC‐derived EVs, HCC‐derived EVs as a biomarker, and the evidence of therapeutic EVs from currently available reports. HCC cells secrete EVs that can lead to increased tumor cell proliferation, migration, chemoresistance, and decreased tumor cell apoptosis. They can also affect tumor microenvironments such as increased angiogenesis. Some of these HCC‐derived EVs can be detected in circulation, making them available for use as a diagnostic biomarker. Moreover, it is possible for EVs to be used as a therapeutic cargo to transfer therapeutic molecules into tumor cells. Several in vitro and in vivo reports have demonstrated antitumoral effects using this method. miR: microRNA; Circ: circular RNA; siRNA: signal interference RNA; ANGPT2: angiopoietin2; linc: long interceding/intergenic noncoding RNA; TUC: tumor ultraconserved RNA.

HCC is common cancer with a high mortality rate. Clinical outcomes of HCC patients have been improved in the past decade due to early tumor detection and the availability of multiple treatment modalities. Currently, although HCC surveillance using ultrasound and serum AFP, imaging‐based diagnosis (CT, MRI), and the new therapeutic methods such as local ablation and transarterial chemoembolization have been shown to be beneficial in increasing survival time, recent data show that the sensitivity of HCC surveillance is still low (about 60%) and that some HCC patients had impaired liver function contradictory to undergoing TACE.^11^ Accumulating evidence shows that HCC cells can use EVs for cell‐to‐cell communication to promote their growth. Clinical studies have already demonstrated that HCC derived‐EV containing RNA cargo can be potential serum biomarkers which may help in the diagnosis of HCC. Some of this EV‐RNA is also associated with tumor burden, indicating that it might be used as a prognostic indicator. In addition, reports from in vitro and in vivo studies indicated that EVs could be used to decrease tumor growth and tumor size. A summary of potential roles of EVs is shown in Figure 1.

This increasing evidence suggests the potential translational use of EVs in HCC patients in the near future. In the case of diagnostic roles, circulating EVs might be used as a serum biomarker for HCC or as an added value for the diagnosis of liver mass with uncertain imaging. Correlating the EVs with tumor staging, tumor size, and metastasis may help to determine tumor burden and prognosis. However, as regards the therapeutic role, there are no clinical reports available at this time. Further studies are needed to discover the best way to use EV for HCC patient care. Investigations aiming to deliver therapeutic EVs via endovascular methods such as using transarterial chemoembolization (TACE) will also provide important information and warrants further clinical investigation.

## ETHICS STATEMENT

Not applicable.

## CONFLICT OF INTEREST

All authors declare that they have no conflict of interest to disclose.

## Data Availability

All information is provided in the manuscript.
